# The Timing and Duration of Folate Restriction Differentially Impacts Colon Carcinogenesis

**DOI:** 10.3390/nu14010016

**Published:** 2021-12-21

**Authors:** Ali M. Fardous, Safa Beydoun, Andrew A. James, Hongzhi Ma, Diane C. Cabelof, Archana Unnikrishnan, Ahmad R. Heydari

**Affiliations:** 1 Department of Nutrition and Food Science, Wayne State University, Detroit, MI 48202, USA; aj9610@wayne.edu (A.M.F.); safab@umich.edu (S.B.); andew.james2@wayne.edu (A.A.J.); al258@wayne.edu (H.M.); diane.cress@wayne.edu (D.C.C.); 2 Department of Molecular and Integrative Physiology, University of Michigan, Ann Arbor, MI 48109, USA; 3 Department of Biochemistry and Molecular Biology, University of Oklahoma Health and Science Center, Oklahoma City, OK 73104, USA; Archana-Unnikrishnan@ouhsc.edu; 4 Barbara Ann Karmanos Cancer Institute, Wayne State University, Detroit, MI 48202, USA

**Keywords:** folate, folic acid, restriction, depletion, colon, cancer, CRC, C57bl/6, mice, mTOR

## Abstract

Diet plays a crucial role in the development of colorectal cancer (CRC). Of particular importance, folate, present in foods and supplements, is a crucial modulator of CRC risk. The role of folate, and, specifically, the synthetic variant, folic acid, in the primary prevention of CRC has not been fully elucidated. Animal studies varied considerably in the timing, duration, and supplementation of folates, leading to equivocal results. Our work attempts to isolate these variables to ascertain the role of folic acid in CRC initiation, as we previously demonstrated that folate restriction conferred protection against CRC initiation in a β-pol haploinsufficient mouse model. Here we demonstrated that prior adaptation to folate restriction altered the response to carcinogen exposure in wild-type C57BL/6 mice. Mice adapted to folate restriction for 8 weeks were protected from CRC initiation compared to mice placed on folate restriction for 1 week, irrespective of antibiotic supplementation. Through analyses of mTOR signaling, DNA methyltransferase, and DNA repair, we have identified factors that may play a critical role in the differential responses to folate restriction. Furthermore, the timing and duration of folate restriction altered these pathways differently in the absence of carcinogenic insult. These results represent novel findings, as we were able to show that, in the same model and under controlled conditions, folate restriction produced contrasting results depending on the timing and duration of the intervention.

## 1. Introduction

Colorectal cancer (CRC) is the second most common reported cancer in western societies. Diet plays a crucial role in the risk and development of CRC. Notably, folates, contained in vegetables and leafy greens, have been associated with a protective effect. Folate deficiency is implicated in the pathogenesis of multiple diseases and conditions such as anemia, cardiovascular disease, neural tube defects, neurodegenerative diseases, and cancer [1]. However, over the past 2 decades, there have been a multitude of reports by independent groups that challenged our understanding of the role of dietary folate in cancer initiation and progression. These reports have been thoroughly reviewed elsewhere [2,3,4,5,6,7,8,9]. Studies have linked inadequate folate consumption with an increased risk of cancer, specifically colorectal cancer (CRC) [10,11,12,13,14]. Others have reported that folate restriction can inhibit colorectal tumorigenesis [15,16,17,18]. The inconsistencies in the published results made it difficult to draw a clear conclusion regarding the effect of folate status on colorectal cancer. The absence of a standard animal model, variations in dietary interventions, and variations in study designs created challenges in drawing parallels across studies. An overview of rodent studies examining the impact of folic acid on CRC revealed that the interventions differed in the use of animal models, diets, folic acid supplementation, antibiotic use, and the type of caging [12,13,14,19,20,21,22,23,24,25]. Of particular importance, the timing, duration, and type of dietary interventions varied considerably, which make comparisons across studies nearly impossible. In most studies, folate-restricted groups received diets with 0 mg/kg folate, while supplemented groups received diets with varying concentrations ranging for 2 mg/kg to 20 mg/kg of folate. The duration of folate restriction, as well as the use of antibiotics and wire-bottom cages to reduce the microflora and coprophagy of the supplied folates, respectively, impacts the serum and tissue-specific folate levels. Comparisons are further complicated by the varying responses to folate restriction when comparing rats to mice, as well as between various genotypes of the same species. A comparative analysis of the experimental design and endpoints of these studies is reviewed elsewhere [26].

To fully characterize the effect of folate on CRC initiation, we must first attempt to define what constitutes a depleted, adequate, or supplemented status in lab models, and how that could translate into human studies. Surprisingly, the folate requirement in rodents is not well characterized. Early mouse studies estimated the minimal folate requirement to be around 0.5 mg/kg diet [27]. Modern rodent diets add a minimum of 2 mg/kg diet folic acid to the standard formulations, while chow diets contain variable amounts of folic acid ranging from 2–15 mg/kg diet, averaging about 8 mg folic acid/kg diet [28]. It is inviting to suggest that the relatively high levels of synthetic folate supplementation to lab rodents in the form of folic acid could modulate cancer risk and confound experimental results. Compared to natural folates, the use of the highly bioavailable folic acid can lead to detectable levels of its unmetabolized form, bypassing some regulations in the one carbon cycle and impacting health and disease differently [29,30]. Another important consideration is that coprophagy and bacterial gut fermentation can supplement the animals with significant folate, even in the absence of dietary sources [31]. The use of sulfonamide antibiotics such as succinyl sulfathiazole (SST) to limit host folate production leads to large shifts in the gut microbiota, further complicating mechanistic analyses by impacting major nutrient sensing, metabolic pathways, and growth pathways [32]. SST reduces folate intake by inhibiting folate-producing bacteria in the gut, thus inducing a severe depletion. We recently revealed that SST can potentially confound results by impacting critical pathways such as mTOR, independently of folate [32].

We previously discovered that a folate restricted diet in a β-pol haploinsufficient mouse model conferred protection against CRC initiation as seen in reduced incidences of the preneoplastic lesions, aberrant crypt foci (ACF). Folate restriction decreased proliferation and increased apoptosis in the intestinal epithelium and decreased the expression of the mammalian target of rapamycin (mTOR) [33]. Hanley et al. used an *Apc*-mutant mouse model to show that a diet deficient in methyl donors can suppress intestinal adenoma by affecting key metabolic pathways [16,34]. Similarly, Lawrance et al. demonstrated that low dietary folate and methylenetetrahydrofolate reductase deficiency were protective in *Apc*^Min/+^ mice [15]. In another model, a folate and choline deficient diet reduced tumorigenesis in serine hydroxy methyltransferase 1 (*Shmt1*) transgenic mice, independently of their genotype [35]. These published reports were conducted in transgenic models that were specifically altered to be predisposed to the development of CRC.

We wanted to test whether folate restriction alone is protective in a wild-type background using a chemically induced cancer model, and to attempt to elucidate the mechanisms behind it. We designed our investigation to characterize and isolate variables that could potentially confound our analysis. Specifically, we were interested in studying the impact of the timing and duration of folate restriction, as well as the use of antibiotics in rodent studies, on tumorigenesis.

## 2. Materials and Methods

### 2.1. Study Design

We conducted a pilot study to determine the effect of a folate-restricted diet on serum folate levels at various timepoints. Baseline serum folate levels of six C57BL/6 mice on a folate adequate diet (2 mg FA/kg diet) were initially assessed. Mice were then placed on a folate-restricted diet (0 mg folic acid/kg) and periodic measurements of serum folate levels were collected from blood samples obtained though orbital venous sinus bleeding. Results are shown in (Supplementary Figure S1).

For the main study, 60 4-month-old male C57BL/6NCrl wild-type mice on a chow diet were acclimated for 2 weeks on an AIN93G semi-synthetic diet. Mice were then randomized into two arms, one receiving the diet containing the antibiotic SST (*n* = 30), and the other without (*n* = 30). Each arm was further randomized across three dietary interventions: full access (FA = folate adequate diet, *n* = 10), delayed restriction (FA/FR = folate adequate diet followed by folate restriction, *n* = 10), and full restriction (FR = folate-restricted diet, *n* = 10). Prior to the carcinogenic treatment initiation, the FA group received a folate adequate diet (2 mg FA/kg diet for 8 weeks), FA/FR received 2 mg FA/kg diet for 7 weeks, then 0 mg folic acid/kg diet for 1 week, and FR received a folate-restricted diet (0 mg folic acid/kg diet for 8 weeks). Each dietary group was further split into untreated controls (CONT) (*n* = 3) and experimental groups, which were treated with the liver and colon cancer initiator dimethylhydrazine (DMH) (*n* = 7). The treatment groups were subjected to a weekly carcinogenic induction using intraperitoneal injections of DMH for 6 weeks followed by a 7-week wait for cancer initiation. All groups were then sacrificed, and their tissues were collected for analyses. A group schematic is presented in Supplementary Figure S2a. The colon form SST treated mice were used for ACF scoring, but liver tissues from the same animals were omitted from the mechanistic analysis of this paper due to the confounding effect of the microbiome perturbation on analyzed pathways, as discussed earlier [32].

### 2.2. Animals

C57BL/6NCrl wild-type male specific pathogen free mice were purchased from Charles Rivers (Wilmington, MA, USA) at 6 weeks of age. Animals were maintained in accordance with NIH guidelines for the use and care of laboratory animals. The animal protocol was approved by the Wayne State University Animal Investigation Committee. Mice were fed the standard mouse chow and water ad libitum and were maintained on a 12 hrs light/dark cycle. Mice were anesthetized in a CO_2_ chamber and the abdominal cavity was opened for excising the colon and harvesting the liver tissue. Blood was obtained via cardiac puncture, centrifuged, and the serum separated. The harvested liver was flash frozen and stored in liquid nitrogen.

### 2.3. Diets

AIN93G-purified isoenergetic diets were made into four standard formulations and were stored at −20 °C (Dyets, Lehigh Valley, PA, USA). The diets either contained no folates (0 mg folic acid/kg diet), no folates with antibiotics (0 mg folic acid/kg diet + 1% SST), a folate adequate diet (2 mg folic acid/kg diet), and a folate adequate diet with antibiotics (2 mg folic acid/kg diet + 1% SST). To avoid over-supplementation in our main study, we used the standard recommended folic acid amount of 2 mg/kg for the AIN−93G diet in our folate adequate groups (FA) [13].

### 2.4. Food Intake and Weights

Animal weights and food intakes were monitored throughout the experiment. The food intake was measured by averaging the food consumed to the number of mice per cage per week.

### 2.5. Carcinogen Treatment

After 8 weeks on their respective diets, mice were injected intraperitoneally with 1, 2-dimethylhydrazine HCl (DMH, 30 mg/kg body weight) in 10 mmol/L of NaHCO_3_ (Fisher Scientific, Pittsburgh, PA, USA) once a week for 6 weeks. Mice were monitored for signs of toxicity and remained on their respective diets for an additional 7 weeks before sacrifice.

### 2.6. Aberrant Crypt Foci (ACF) Analysis

The excised colons were rinsed with cold phosphate-buffered saline, cut longitudinally, and fixed flat overnight in 10% neutral buffered formalin. The fixed colons were stained with 2 g/L of methylene blue in phosphate-buffered saline for 5 min. The number of ACF and aberrant crypts per foci were determined by light microscopy at 100× magnification in a blinded manner, as described previously [33].

### 2.7. Folate Assay

Serum, liver, and colon mucosa were collected upon sacrifice. Folate was measured using the *Lactobacillus casei* microbiological assay of folic acid derivatives, as described previously [16]. The growth response of *Lactobacillus casei* compared to folate availability was measured at OD 600 nm. A standard curve was used to calculate folate concentrations. Folate levels were expressed as nmol/g for liver and colon tissues and fmol/uL for serum.

### 2.8. Isolation of Whole Cell Extract and Sample Preparation for Western Blotting

Fifty mg of liver tissue was homogenized using a RIPA buffer (1% Igepal, 0.5% sodium deoxycholate, 0.1% SDS, 1X PBS) combined with a Halt protease inhibitor cocktail (Thermo Fisher, Waltham, MA, USA). Protein concentrations were quantified using the Pierce BCA protein assay (Thermo Fisher, Waltham, MA, USA). Volumes were normalized to 50 ug of total protein per sample for Western blotting and were aliquoted into multiple sets with equivalent volumes of LDS sample loading, then were stored at −80 °C.

### 2.9. Western Blot Analysis

A protein expression analysis was performed using the Bio-Rad V3 Western blot system. Samples were denatured by boiling and a normalized volume, equivalent to 50 μg of protein per well, was loaded onto 2 criterion TGX stain free precast gels along with Precision Plus Protein unstained standards (Bio-Rad, Hercules, CA, USA). Gels were electrophoresed at 300 volts in the same apparatus. Both gels were then UV activated and the stain free image was captured using the ChemiDoc XRS+ System (Bio-Rad, Hercules, CA, USA). Imaged gels were wet transferred together in the same apparatus onto nitrocellulose membranes at 100 volts for 30 min. Membranes and gels were imaged to verify the complete transfer. The same exposure settings were used for all gels and membranes during the study to ensure consistency. Using these defined exposure settings, a linear regression was calculated using a serial protein dilution, which was blotted and analyzed through Image Lab Software (Bio-Rad, Hercules, CA, USA). The resulting formula was used to calculate and normalize total lane proteins for our experimental runs across both gels. Membranes were blocked with 5% skim milk in TBS for 1 h. Both membranes were incubated together with primary antibodies using the manufacturer’s recommended dilution of 1xTBS with 5% BSA overnight on a tilting shaker at 4 °C. A list of antibodies is provided in the Supplementary Table S3. Membranes were then washed five times in TBST, followed by their incubation with an HRP-conjugated secondary antibody (Santa Cruz Biotechnology, Santa Cruz, CA, USA). The resulting bands on both membranes were exposed simultaneously and were quantified using the ChemiDoc XRS+ imager and Image Lab Software after incubation in a SuperSignal West Pico chemiluminescent substrate (Pierce Biotechnology, Rockford, IL, USA). Data are expressed as adjusted band volumes that are normalized to total lane proteins, as described by Taylor et al. [36]. Phosphorylated forms of proteins were blotted and quantified first, then the membrane was lightly stripped and incubated with their respective total protein antibody after the verification of successful stripping. Phosphorylated forms of proteins were normalized to totals and expressed as ratios. Western blot images are provided in the Supplementary Table S1.

### 2.10. Gene Expression Profiling

Total RNA was extracted from liquid nitrogen flash frozen liver tissue using the RNeasy extraction kit (Qiagen, Valencia, CA, USA). First strand cDNA was synthesized from 1 μg RNA using random primers and poly-A primers. The expression of each gene was quantified by real-time PCR using pre-designed and validated gene-specific primers. The relative expression was calculated using the ΔΔCT method and was normalized to the geometric mean of *Gapdh* and *Rplp0*. Primer sequences are provided in Supplementary Table S2.

### 2.11. ATP Assay

Ten mg of liver tissue was homogenized with a nucleotide releasing buffer and the ADP/ATP ratio was measured by bioluminescence, as per the manufacturer’s protocol (Abcam, Cambridge, UK).

### 2.12. NAD^+^/NADH Assay

Twenty mg of liver tissue was homogenized in a provided lysis buffer and the supernatant was collected. Total NAD, NAD^+^, and NADH were measured through fluorescence (EX/EM = 540/590 nm) as per the manufacturer’s protocol (Abcam, Cambridge, UK).

### 2.13. Statistical Analysis

The statistical significance between means was determined using a one-way and two-way ANOVA followed by a post-hoc Tukey test wherever appropriate (GraphPad Software, CA, USA). *p*-values less than 0.05 were considered statistically significant.

## 3. Results

### 3.1. Effects of Folate Restriction on the Folate Level and Weight

**Figure 1:**
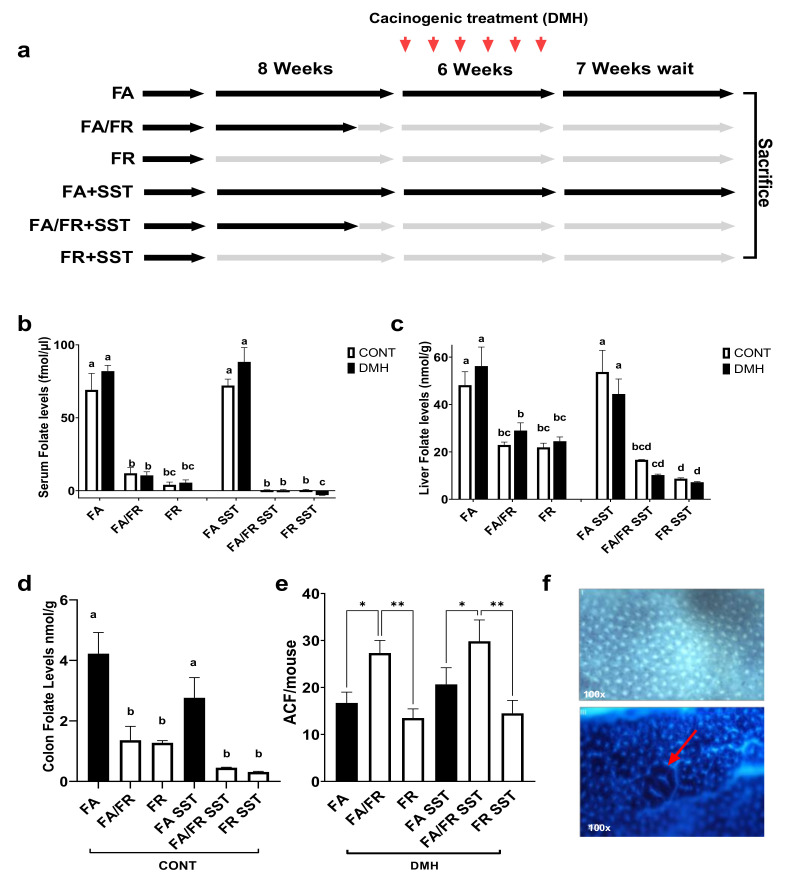
FR reduces ACF formation in DMH-treated male C57BL/6 mice compared to FA/FR, irrespective of SST treatment. (**a**) Study design. (**b**) Effect of FA/FR +/− SST and FR +/− SST on serum folate levels. (**c**) Effect of FA/FR +/− SST and FR +/− SST on liver folate levels. (**d**) Effect of FA/FR +/− SST and FR +/− SST on colon folate levels in untreated controls. (**e**) Effect of FA/FR +/− SST and FR +/− SST on colon ACF formation in DMH-treated mice. (**f**) Top image is a stained colon displaying normal crypts, bottom image shows a raised ACF in the center (red arrow). FA = folate adequate diet, FA/FR = folate adequate diet followed by initiation of folate-restricted diet one week prior to DMH injections, FR = folate-restricted diet. SST = 1% succinylsulfathiazole added to diet; CONT= control; no carcinogenic treatment, *n* = 3; DMH = dimethylhydrazine, a liver/colon tumor initiator, *n* = 6–7. Bars, S.E.M, means with different letters are significantly different (Tukey’s HSD, *p* < 0.05), * *p* < 0.05, ** *p* < 0.005.

Results from our pilot study revealed a rapid and significant serum folate level reduction after one week of folate restriction, followed by stabilizing thereafter. No further reduction was observed at 12 weeks (Supplementary Figure S1). Due to the likelihood of coprophagy and bacterial folate intake through gut fermentation, we termed the condition folate restriction (FR) instead of folate depletion. Folate levels were assayed 7 weeks after the last DMH injection at the time of animal sacrifice. A timeline of the study is provided in (Figure 1a). There was an equal reduction in folate levels between FA/FR and FR mice in serum, liver, and colon samples. SST further reduced detectable folate levels in both folate-restricted groups compared to non-SST treated mice; however, the observed reduction did not always reach statistical significance (Figure 1b–d). The analysis of folate levels in our folate-restricted control groups revealed a significant reduction of serum, liver, and colon folates (FA/FR: 83%, 53%, and 68%), (FR: 95%, 55%, and 70%), (FA/FR SST: 99%, 69%, and 84%), (FR SST: 99%, 84%, and 89%), respectively. A table depicting the average measured folate levels is provided in Supplementary Table S4. Food intake did not vary significantly between groups throughout the experiment. We did not observe any difference in weights between matched groups (Supplementary Figure S2b–e). There was no significant difference in weight across all groups at the endpoint of the experiment (Supplementary Figure S2f).

### 3.2. Adaptations to Folate Restriction Protects against ACF Formation

To determine how the timing of folate restriction impacts colon carcinogenesis, the colons of mice treated with DMH were screened for the development of aberrant crypt foci (ACF), a preneoplastic lesion representing the initial stages of cancer initiation and development. Placing the animals on a folate-restricted diet for one week (FA/FR) before initiating the carcinogenic insult resulted in significantly more ACF compared to animals that were fed a folate-supplemented diet. However, mice placed on 8 weeks of a folate-restricted diet (FR) developed significantly less ACF compared to FA/FR (Figure 1e). The same pattern was observed in SST supplemented groups, with FR SST mice developing significantly less ACF than FA/FR SST (Figure 1e).

### 3.3. Folate Restriction Modulates the mTOR Pathway

**Figure 2:**
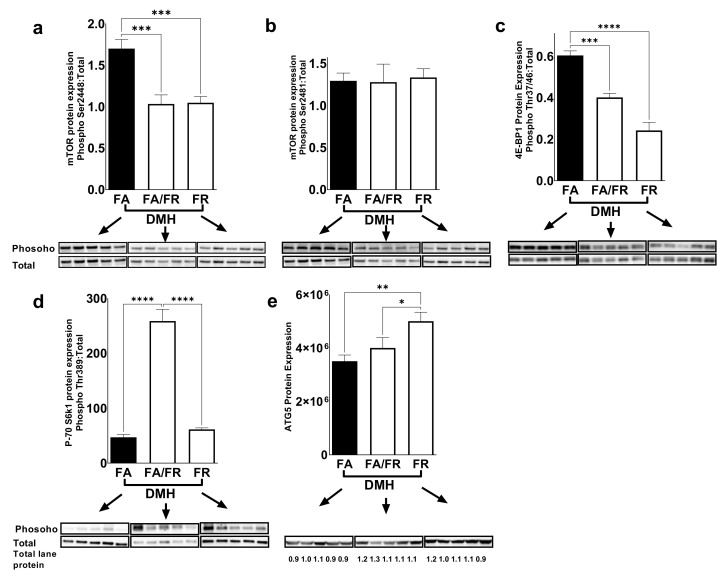
Folate restriction downregulates the mTOR signaling pathway in the liver of DMH-treated mice. Western blot images and quantification of (**a**,**b**) mTOR phosphorylation at Ser2448 and Ser2481 residues, and (**c**,**d**) phosphorylation of downstream targets of mTOR (4EBP1 (Thr37/46)) and S6K1 (Thr389), and (**e**) expression of the autophagy related protein ATG5. FA = folate adequate diet, FA/FR = folate adequate diet followed by initiation of folate-restricted diet one week prior to DMH injections, FR = folate-restricted diet. Phosphorylated protein was normalized to the respective total protein unless indicated and expressed as ratio. Bands were normalized to total lane proteins. DMH = dimethylhydrazine, liver/colon carcinogenic treatment, *n* = 5. Bars, S.E.M, * *p* < 0.05, ** *p* < 0.005, *** *p* < 0.001, **** *p* < 0.0001.

mTOR is a conserved serine/threonine protein kinase that integrates signals such as growth factors, amino acids, energy levels, and nutrient availabilities to modulate cell growth, proliferation, autophagy, and metabolism. We were interested in investigating the mTOR pathway as a potential target of folate restriction that can modulate CRC risk. mTORc1 is activated via phosphorylation at Ser2448 via the IGF/PI3K/AKT signaling pathway, and we observed reduced phosphorylation in both FA/FR DMH and FR DMH conditions (Figure 2a). No difference was observed at the auto-phosphorylation site of mTORc1 at Ser2481 (Figure 2b). mTORc1 regulates protein synthesis and growth through the downstream targets, 4E-BP1 and S6K1. 4E-BP1 phosphorylation at Thr37–46 was significantly decreased in FR DMH compared to both FA/FR DMH and FA DMH (Figure 2c). We observed increased phosphorylation of S6K1 at Thr389 in FA/FR DMH compared to FA DMH and FR DMH (Figure 2d). ATG5, a key autophagy related protein that is downstream of mTOR was significantly upregulated in FR DMH only (Figure 2e). In the absence of carcinogenic insult, the duration of folate restriction (21 weeks for FR vs. 14 weeks for FA/FR) modulated the mTOR pathway differently compared to DMH-treated animals. We noted that in the control groups, FA/FR downregulated the mTOR pathway as evident in decreased mTOR, 4E-BP1, and S6K1 phosphorylation compared to FA, while FR had no effect (Supplementary Figure S3). There was no difference in the protein expression of ATG5 between the control groups (Supplementary Figure S3).

**Figure 3:**
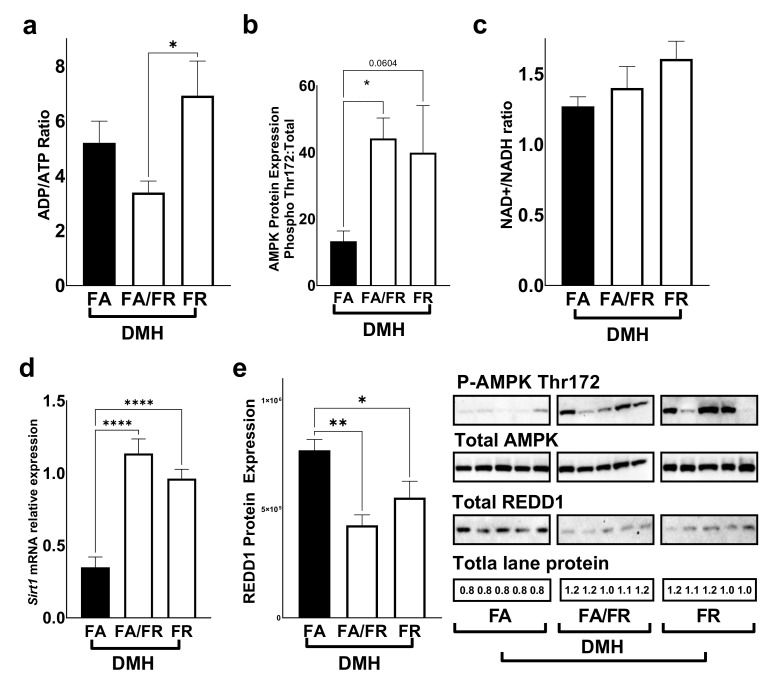
Folate restriction alters energy levels and regulators of mTOR in the liver of DMH-treated mice. (**a**) ADP/ATP ratio in the liver. (**b**) Western blot quantification of AMPK (Thr172) protein phosphorylation. (**c**) NAD+/NADH ratio (**d**). Sirt1 mRNA relative expression. (**e**) Western blot quantification of REDD1 protein levels. FA = folate adequate diet, FA/FR = folate adequate diet followed by initiation of folate-restricted diet one week prior to DMH, FR = folate-restricted diet. Western blots were normalized to total lane proteins. DMH = dimethylhydrazine, liver/colon carcinogenic treatment; *n* = 3 for energy assays; *n* = 5–7 for western blots and qPCR analysis; Bars, S.E.M, * *p* < 0.05, ** *p* < 0.005, **** *p* < 0.0001.

We used the ADP/ATP ratio as an indicator of energy availability in liver tissues. We saw a decrease in the ADP/ATP ratio in FA/FR DMH compared to FR DMH, indicating increased energy availability (Figure 3a). AMPK, a critical regulator of mTORc1, is activated through phosphorylation at Thr172 as it detects changes in the intracellular AMP/ATP ratio. FA/FR DMH experienced a significant increase in AMPK phosphorylation compared to FA DMH, while FR-induced AMPK activation did not reach statistical significance (*p* = 0.06) (Figure 3b). The further analysis of energy levels in the form of the NAD+/NADH ratio did not reveal a significant difference between our experimental groups (Figure 3c). SIRT1, a well-characterized NAD+ dependent deacetylase and a negative regulator of mTOR, experienced an increase of mRNA expression in both FA/FR DMH and FR DMH, compared to FA DMH (Figure 3d). The analysis of REDD1, a marker of hypoxia, DNA damage, and a negative regulator of mTORc1, revealed attenuated protein expressions in both FA/FR and FR DMH-treated animals as compared to FA DMH (Figure 3e). The analysis of control animals that were not exposed to DMH revealed a similar pattern to the DMH-treated mice. We noted an increase in the ADP/ATP ratio in FR compared to FA/FR, translating into an activation of AMPK in the FR controls (Supplementary Figure S4). No significant difference was observed in the NAD+/NADH ratio, *Sirt1* gene expression, or REDD1 protein expression between the control groups (Supplementary Figure S4).

### 3.4. Folate Restriction Reduces the Expression of Methylation Genes

**Figure 4:**
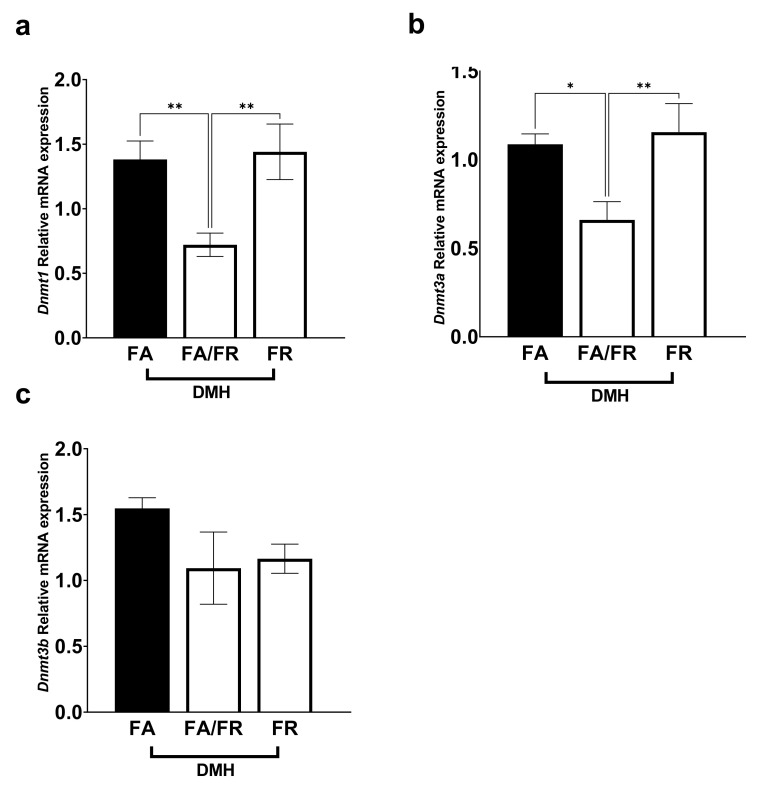
Folate restriction alters DNA methyltransferase expression in the liver of DMH-treated C57BL/6 mice. (**a**) *Dnmt1* relative mRNA expression. (**b**) *Dnmt3a* relative mRNA expression. (**c**) *Dnmt3b* relative mRNA expression. Real-time expression data was normalized to geometric mean of *Gapdh* and *Rplp0*. FA = folate adequate diet, FA/FR = folate adequate diet followed by initiation of folate-restricted diet one week prior to DMH injections, FR = folate-restricted diet. *n* = 6–7, Bars, S.E.M, * *p* < 0.05, ** *p* < 0.005.

DNA methyltransferases are involved in generating and maintaining CpG methylation across the genome. *Dnmt1* expression decreased significantly in FA/FR DMH, compared to FA DMH and FR DMH (Figure 4a). Similarly, *Dnmt3a* mRNA levels significantly decreased in FA/FR DMH, but not FR DMH, compared to FA DMH (Figure 4b). The expression of *Dnmt3b* was not significantly impacted by the folate status of DMH-treated animals (Figure 4c). The analysis of DNA methyltransferases in the untreated control mice revealed different results, where FR resulted in a decreased expression of all three methyltransferases (this was significant for *Dnmt3a* and *Dnmt3b*, with a downward trend in *Dnmt1*) (Supplementary Figure S5).

### 3.5. Folate Restriction Impacts DNA Repair, Antioxidants, and Tumor Suppressor Genes

**Figure 5:**
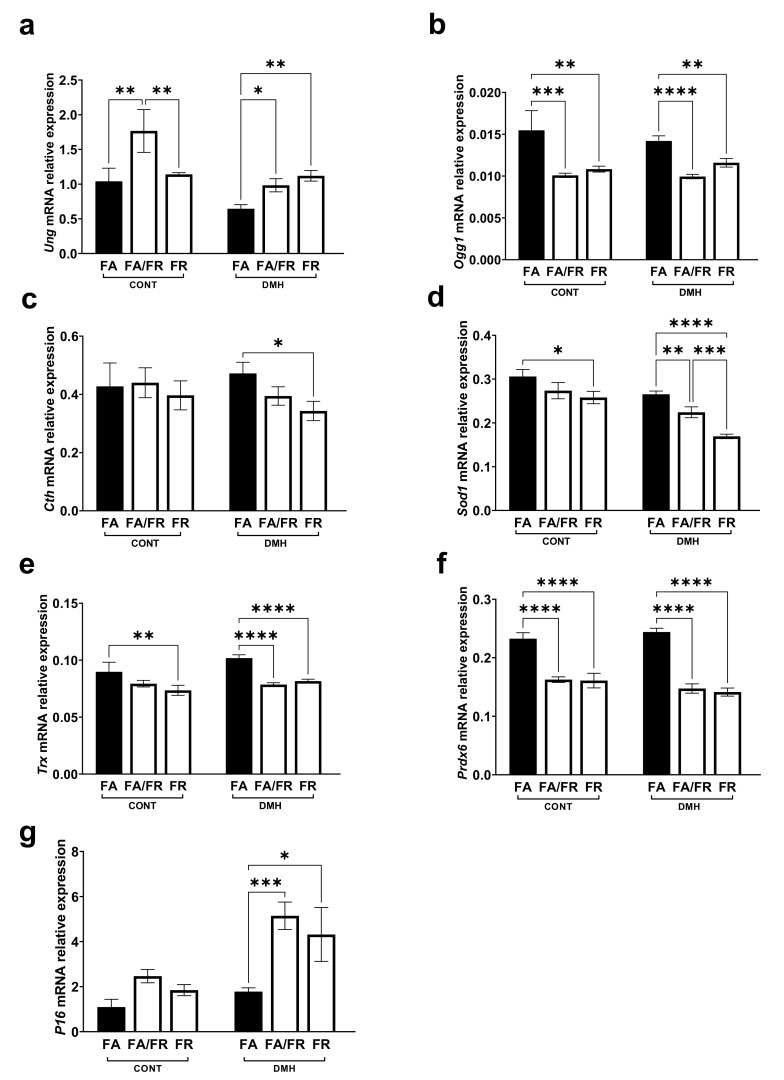
Effect of the timing and duration of folate restriction on relative expression of genes known to be altered by folate status and oxidative stress in the liver of untreated controls (CONT) and dimethylhydrazine treated (DMH) c57BL/6 mice. (**a**,**b**) mRNA relative expression of uracil DNA glycosylase (*Ung*) and 8-oxoguanine DNA glycosylase (*Ogg1*). (**c**) mRNA relative expression of cystathionine-γ-lyase (*Cth*). (**d–f**) mRNA relative expression of the antioxidant genes superoxide dismutase (*Sod1*), thioredoxin (*Trx*), and peroxiredoxin (*Prdx6*). (**g**) mRNA relative expression of the tumor suppressor *P16 ink4a*. FA = folate adequate diet, FA/FR = folate adequate diet followed by initiation of folate-restricted diet one week prior to DMH injections, FR = folate-restricted diet, CONT = untreated control mice, *n* = 3; DMH = mice treated with the liver/colon carcinogen dimethylhydrazine, *n* = 6–7. Real-time data were normalized to geometric mean of *Gapdh* and *Rplp0*. Bars, S.E.M, * *p* < 0.05, ** *p* < 0.005, *** *p* < 0.001, **** *p* < 0.0001.

The expression of DNA repair genes, such as *Ung* (uracil DNA glycosylase), was upregulated, while *Ogg1* (8-oxoguanine DNA glycosylase) was downregulated in FA/FR DMH and FR DMH compared to FA DMH (Figure 5a,b). Cystathionine-γ-lyase (*Cth*), the enzyme that converts cystathionine to cysteine, is a critical regulator of the transsulfuration pathway. We observed that FR reduced the expression of *Cth* in DMH-treated animals (Figure 5c). To assess that impact of folate on major antioxidant regulators, we looked at the gene expression of superoxide dismutase (*Sod1*), thioredoxin (*Trx*), and peroxiredoxin (*Prdx*). While *Sod1* gene expression was significantly reduced in both FA/FR DMH and FR DMH animals compared to FA DMH, FR DMH experienced a significantly more pronounced decrease (Figure 5d). FA/FR DMH and FR DMH animals experienced a similarly significant decrease in *Trx* and *Prdx* gene expression compared to FA DMH (Figure 5e,f). The expression of *P16 ink4a*, a cyclin-dependent kinase inhibitor, tumor suppressor, and a downstream target of SIRT1, was increased in FA/FR DMH and FR DMH compared to FA DMH (Figure 5g). The analysis of the genes discussed above in untreated control groups produced results that mostly mirrored what we observed in the respective DMH groups (Figure 5a–g). However, FR induced a reduction of *Ung* gene expression compared to FA/FR (Figure 5a), while no significant difference between the control groups was observed in the analysis of *Cth* and *P16* (Figure 5c,g).

## 4. Discussion

Studies investigating the effect of folate restriction have typically employed lab rodents using chemically induced, or spontaneous, models of CRC. Our results revealed that placing the mice on a folate-restricted diet significantly reduced serum and tissue folate levels (83–99% reduction in serum folate). Similarly, studies using 2 mg folic acid/kg diet as folate adequate groups reported a 50–96% reduction in blood folate levels compared to a folate-restricted diet of various durations [12,13,14,19,22,24,37]. An important observation is that many of these studies resorted to the use of antibiotics or the prevention of coprophagy to induce a significant folate depletion, as measured by a reduction of blood folate exceeding 80% [14,19,22,37]. We were able to achieve comparable levels of folate depletion irrespective of the use of antibiotics and without coprophagy prevention. Consistent with these previously published reports, our use of SST did induce a more severe depletion, with folate levels falling below the detectable threshold in the serum, while we observed a downward trend in the liver and colon tissues compared to non-SST groups. Furthermore, at the time of sacrifice, the difference in the total duration of folate restriction between FA/FR and FR (14 weeks vs. 21 weeks total), with and without SST, did not lead to an additional decrease in folate levels. This is consistent with our previous finding that mice required about 8 weeks of folate restriction to become severely depleted [37]. An important consideration when comparing plasma and tissue folate level reductions in folate restriction studies is the method of detection. The *L. casei* microbiological assay was used in this study. However, methods of folate detection, such as LC–MS/MS, could produce more accurate and specific measurements of folate and its metabolites in serum and tissues, while being less susceptible to interference from antibiotics and contaminants [38,39].

It is accepted that folate deficiency in tissues can lead to uracil misincorporation due to the decrease in thymidylate synthesis, causing DNA strand breaks, impaired repair, and genomic instability [26,40]. Furthermore, folate deficiency can lead to aberrant DNA methylation due to the decrease of the universal methyl donor S-adenosylmethionine (SAM). A folate adequate diet is expected to correct these aberrations, leading to a decrease in cancer risk. However, in preneoplastic or fully transformed lesions, folate restriction can inhibit the growth requirements of a rapidly proliferating tissue, leading to decreased tumor growth and progression, while FA supplementation can provide the tumor with nucleotides, leading to faster growth and aggressiveness [41]. Nevertheless, there is mounting evidence that low folate availability may also protect against spontaneous and chemically induced cancer [16,42]. Epidemiological studies have linked elevated folates with a higher incidence of various cancers, including colon cancer [6,43]. Consistent with most rodent studies, placing our mice on one week of folate restriction (FA/FR) increased ACF formation. However, when mice adapted to folate restriction for 8 weeks (FR), they were protected. The protection was consistent with what we had previously uncovered in our β-pol haploinsufficient mice. Similarly, Macfarlane et al. showed that APC^min^ and *Shmt* mutant mice placed on folate restriction experienced a decrease in the tumor number and size [24]. Another study by Song et al. using the APC^min^ model revealed that folate restriction was protective at 6 months, but not at 3 months [12]. In a chemically induced tumorigenesis rat model, Le Leu et al. found that folate restriction resulted in a three-fold reduction in ACF compared to rats supplemented with 8 mg folic acid/kg diet [18]. The significance of our findings is that it takes place in normal tissues, while other studies have been conducted in mutant models that are predisposed or partially transformed. Genetically predisposed models, such as the APC mutants, acquire precancerous lesions early in their development, so the timing of folate restriction can impact tumorigenesis differently depending on the presence or absence of preneoplastic lesions. These results may not translate well to humans, where colon cancer occurs predominantly as a sporadic event precipitated by age and genotoxic insults, while hereditary etiologies arising from mutations of tumor suppressors such as APC account for only 1–5% of cases [44]. The results of this study reveal that folate restriction can prevent cancer initiation in a wild-type background. Our mice were housed in normal cages with no wire bottoms, leading to the likelihood of coprophagy and potentially providing a minor source of folate in the form of bacterial products. The addition of the antibiotic SST, to induce a severe depletion, did not abrogate the protective effect of FR. This would suggest that severe folate depletion and the perturbation of the microbiome does not significantly impact CRC initiation in this model. Hanley et al. showed that a temporary methyl donor-depleted diet conferred long-lasting protection against intestinal adenoma by inducing changes to the intestinal epithelium [16]. Adapting the animals to folate restriction could potentially induce similarly protective changes. β-pol haploinsufficient mice on folate restriction exhibited reduced proliferation and increased apoptosis in the colon epithelium [33]. Additional research is needed to test if similar protective mechanisms occur in the colon epithelium in wild-type mice.

Our previous work outlining the restriction of folate’s protective effect in the β-pol model revealed that the mTOR pathway was downregulated in the folate-restricted groups [33]. In fact, the IGF/mTOR/S6K1 signaling cascade plays an important role in colorectal carcinoma [45]. mTOR’s central role in integrating signals for growth factors, amino acids, energy levels, and nutrient availability to modulate growth, autophagy, proliferation, and metabolism made it an attractive target for investigation. Others have shown that mTOR is modulated by perturbation in one carbon metabolism and, more specifically, in folate availability in cancer [46]. A folate-restricted diet downregulated mTORc1 in both of our folate-restricted groups, FA/FR DMH and FR DMH, compared to FA DMH. Similarly, folate deficiency in mice inhibited both mTORc1 and mTORc2 signaling in a pregnant mouse model as well as in human trophoblasts [47]. Folic acid can modulate the PI3K/AKT pathway upstream of mTOR, leading to its activation, as well as the activation of the downstream targets S6K1 and 4E-BP1 [48]. Furthermore, both mTOR and the one-carbon cycle are regulated through energy availability. The NAD+/NADH ratio, maintained by mitochondrial oxidative phosphorylation, links energy levels and the one-carbon cycle through formate production from serine, as well as regulating mTOR through SIRT1 [49,50]. Our analysis revealed an upward trend in the NAD+/NADH ratio between the FR DMH and FA DMH groups; however, SIRT1 expression was significantly increased in both FA/FR DMH and FR DMH compared to FA DMH. Research in *C. elegans* revealed that low levels of folic acid supplementation inhibits the mTOR and IGF1 pathways [51]. The activation of the mTOR negative regulators, AMPK and SIRT1, in our folate-restricted DMH-treated groups suggests that mTOR’s inhibition in our model is likely mediated through energy availability in the liver. The analysis of the downstream targets of mTOR, 4E-BP1 and S6K1, revealed a distinct difference between FA/FR and FR in DMH-treated mice. FR decreased 4E-BP more significantly than FA/FR, compared to FA, indicating a more pronounced inhibition on cap-dependent translation. Remarkably, FA/FR induced a significant increase in S6k1 phosphorylation, while FR had no effect. S6K1 activation can potentially be mediated by TOR-insensitive signaling pathways such as PDK1, MAPK, and SAPK [52,53]. The activation of autophagy in FR, but not in FA/FR as seen by the increase in the protein expression of the autophagy marker ATG5, is also of potential significance, confirming that FR impacts the downstream targets of mTOR differently than FA/FR.

Folate status has a direct and significant effect on DNA methylation. Perturbances in the folate pools, stemming from a low or high folate status, can result in hypomethylation or hypermethylation, leading to epigenetic instability [54]. DNA hypomethylation is considered an early event in colon carcinogenesis, causing an increase in gene expression and DNA strand breaks [55]. Folate-restricted diets increase the levels of homocysteine and S-adenosyl-homocysteine (SAH), leading to the inhibition of DNA methyltransferases (DNMT), resulting in global DNA hypomethylation [56,57]. In contrast, folic acid supplementation was linked to an increase in DNMT activity [58]. The effect of folate restriction on methylation was not consistent across studies. A review by Kim et al. reported that folate restriction affected DNA methylation differently, depending on the severity and length of folate restriction, as well as the use of carcinogenic treatments such as DMH or azoxymethane (AOM) [59]. Aberrant methylation alone could not consistently explain how folate restriction may promote carcinogenesis. In our DMH-treated animals, the expression levels of *Dnmt1* and *Dnmt3A* in FR animals were equivalent to that of FA, while in FA/FR, both gene expressions were significantly reduced. This suggests that animals that are adapted to long-term folate restriction, and then exposed to DMH, mimic FA in the expression of critical maintenance methyltransferases. This is consistent with the findings by Song et al. [12] noting an adaptive rebound of genomic DNA methylation after 8 weeks on folate-depleted diets. Works by Eads et al. and Weis et al. reveal that the deletion of *Dnmt1* and *Dnmt3a* prevented tumorigenesis in APC-mutant mice [60,61]. This is in contradiction to our observation, where FA/FR inhibited both *Dnmt1* and *Dnmt3a,* while resulting in a deleterious outcome in our wild-type mice. The differential effect of FR, compared to FA/FR, on DNA methyltransferase expression represents an attractive target for further analyses to fully characterize the impact on global, gene-specific, and tissue-specific methylation patterns.

Folate’s role in the one-carbon cycle leading to DNA synthesis and repair, as well as maintaining a balanced redox state, is well documented. Consistent with our previous findings, folate restriction in combination with an oxidative stressor (DMH) led to an increase in *Ung* expression, while *Ogg1* decreased [37,62,63]. While an increase in the uracil DNA glycosylase *Ung* is expected, resulting from uracil’s misincorporation into DNA under folate restriction, the decrease of 8-oxoguanine glycosylase is perplexing as this enzyme is responsible for removing mutagenic bases resulting from exposure to reactive oxygen species. Furthermore, we noted a reduced expression of *Cth* in FR DMH, potentially suggesting decreased flux through the transsulfuration pathway. Additional analyses confirmed a general reduction in antioxidant potential in both FA/FR and FR, evident through a decrease of SOD1, TRX, and PRDX gene expressions. Our results contradict findings by Hanley et al. where a methyl donor-deficient diet protected against CRC and resulted in increased flux through the transsulfuration pathway, increased glutathione and endogenous antioxidants, and reduced oxidative stress markers in the colon [34]. However, we must note that the mechanism of protection that Hanley et al. observed might be different due to the use of APC-mutant mice and diets deficient in choline, methionine, B12, and folic acid. Combined, our results may suggest that the perturbation of the one-carbon cycle through folate restriction favors nucleotide synthesis and energy metabolism, instead of maintaining the SAM/SAH ratio and the generation of the antioxidant glutathione. We hypothesize that FR’s inhibition of mTOR, 4eBP, and S6k, as well as the upregulation of ATG5 and the cell cycle arrest gene P16, indicate that mice adapted to folate restriction favor reduced proliferation and increased autophagy. Of particular importance, the reduction of DNA methyltransferases observed in the FA/FR DMH animals potentially indicate reduced DNA methylation. While FR DMH animals exhibited comparable DNMT expression to FA DMH animals, this could imply that the kinetics of the one-carbon cycle may be altered as the animals adapt to folate restriction.

Adding another layer of complexity to this analysis is the fact that folate restriction among the control groups that were not exposed to the carcinogenic insult revealed differing patterns of gene and protein expressions compared to the DMH-treated mice. It is important to note that within the control groups, FR mice differed from FA/FR mice in the length of folate restriction only (21 weeks vs. 14 weeks, respectively). Nevertheless, we noted that FA/FR caused the downregulation of mTOR and its downstream targets, 4EBP1 and S6K1, while the continued restriction for an additional 7 weeks in FR mice caused a rebound of the same proteins. Paradoxically, decreased energy levels in the liver of FR controls and the corresponding activation of AMPK contradicts the observed effects on the mTOR pathway. Furthermore, in the absence of DMH, we saw a decrease in gene expression of all three DNA methyltransferases tested, which corresponds to the increased duration of folate restriction in FR vs. FA/FR mice. Taken together, these interactions reveal dynamic changes that occur with time as the animals adapt to folate restriction. These observations provide additional support to the notion that the timing of genotoxic stress can influence outcomes in folate-restricted mice. It is also apparent that DMH treatment influences how adaptations to folate restriction impact critical pathways. A diagram summarizing the effects of folate restriction on the mTOR pathway and DNA methyltransferases in DMH-treated mice is provided in Supplementary Figure S6.

An important consideration for future research is that, in addition to characterizing the nature of the potential beneficial adaptation, as well as validating the results in different genetic backgrounds and models, it is important to determine the time at which that adaptation takes place and whether it persists with prolonged restriction. Our study reveals that the adaptation to folate restriction occurs prior to DMH initiation during the preceding 7 weeks of folate restriction imposed on FR mice, compared to 1 week in FA/FR mice. Future studies can be designed to elucidate the changes that occur during this critical period, as well as to test whether the differential outcomes between FA/FR and FR mice are driven primarily through a beneficial adaptation to low folate status, or if it is partly the result of combining acute folate restriction with carcinogenic insult. Furthermore, it is necessary to test the efficacy of this proposed adaptation not only in the prevention of cancer initiation, but also in preventing adenomas, tumor multiplicity, and metastatic growth.

## 5. Conclusions

Our work provides yet another indication of the increasingly complex effect of folate on cancer. This research demonstrated that adapting mice to folate restriction can be protective against colon cancer initiation. We showed that the timing and duration of folate restriction differentially impacted critical pathways, such as mTOR, methylation, DNA repair, and oxidative stress. While more research is needed to fully unravel the underlying dynamics of this observed protection, our results provide important leads. Controversies stemming from contradictory animal and human reports continue to challenge our understanding of the role of folate in CRC. Our findings are significant because they could potentially explain the discrepant results observed in many rodent studies. To our knowledge, this is the first report that shows that folate restriction can be both beneficial and deleterious in the same wild-type model. These results lead one to question many of the rigid assumptions we have had about the role of folates in CRC, revealing a much more intricate picture.

## Supplementary Materials

The following are available online at https://www.mdpi.com/article/10.3390/nu14010016/s1, Figure S1: The effect dietary folate restriction on plasma folate levels at various timepoints, Figure S2: Effect of folate restriction on animal weights, Figure S3: Folate restriction alters the expression of proteins in the mTOR signaling pathway in the liver of untreated control C57BL/6 mice, Figure S4: Effect of the timing and duration of folate restriction on energy levels and regulators of mTOR in the liver of untreated control C57BL/6 mice, Figure S5: Folate restriction downregulates DNA methyltransferase gene expression in the liver of untreated control C57BL/6 mice, Figure S6: Summary of the effects of FA/FR and FR on the mTOR pathway and DNA methyltransferases in the liver of DMH treated mice, Supplementary Table S1: Western blots, Supplementary Table S2: Primer sequences, Supplementary Table S3: Antibodies for western blots, Supplementary Table S4: Average measured folate levels in serum, liver and colon.
