# Supplementary material for: The Timing and Duration of Folate Restriction Differentially Impacts Colon Carcinogenesis
Source: Nutrients. 2021 Dec 21;14(1):16. doi: 10.3390/nu14010016 (PMC8746403; doi:10.3390/nu14010016)
Supplement: Supplementary file 1 [file nutrients-14-00016-s001.zip › nutrients-1479498-supplementary materials.pdf]

Supplementary Materials

The Timing and Duration of Folate Restriction Differentially Impacts Colon Carcinogenesis

Ali M. Fardous[1,†], Safa Beydoun[1,2,†], Andrew A. James[1], Hongzhi Ma[1], Diane C. Cabelof[1,] Archana Unnikrishnan[3] and Ahmad R. Heydari[1,4,*]

Supplemental Figure S1

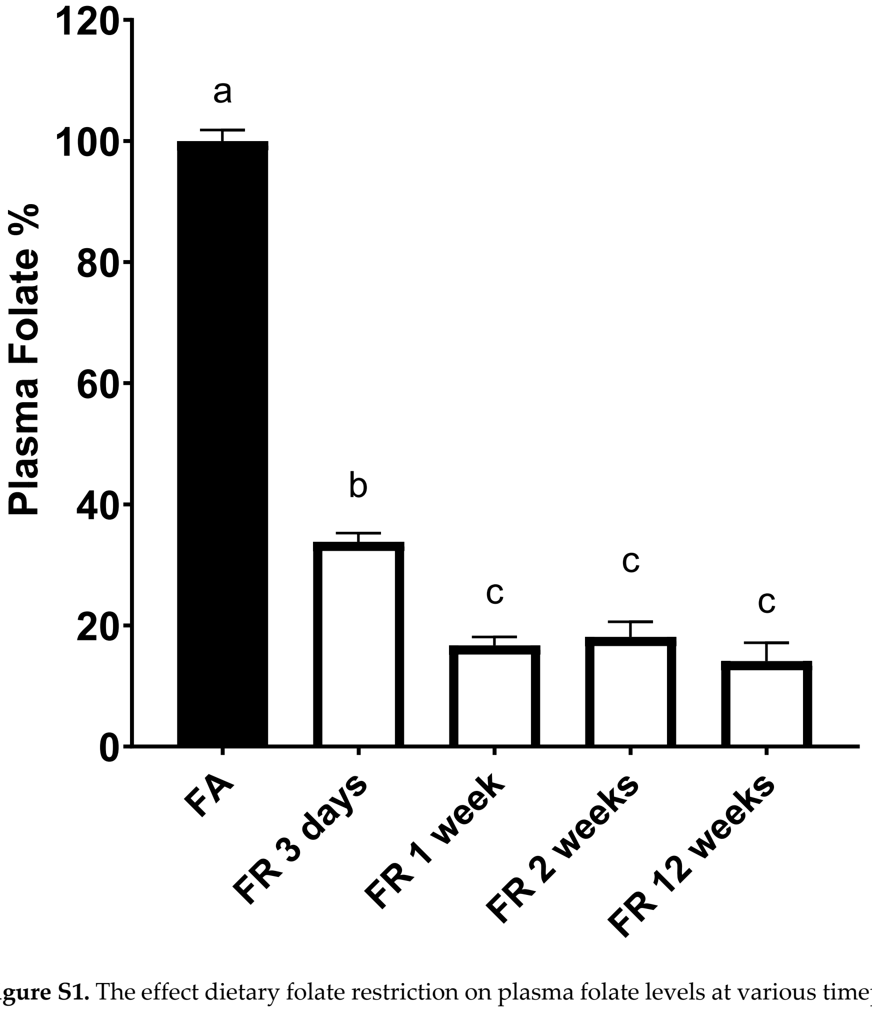

Figure S1. The effect dietary folate restriction on plasma folate levels at various timepoints. (FA=folate adequate diet), (FR= folate restricted diet) Bars, S.E.M. (n=6), different letters denote significance, p<0.0001.

Supplemental Figure S2

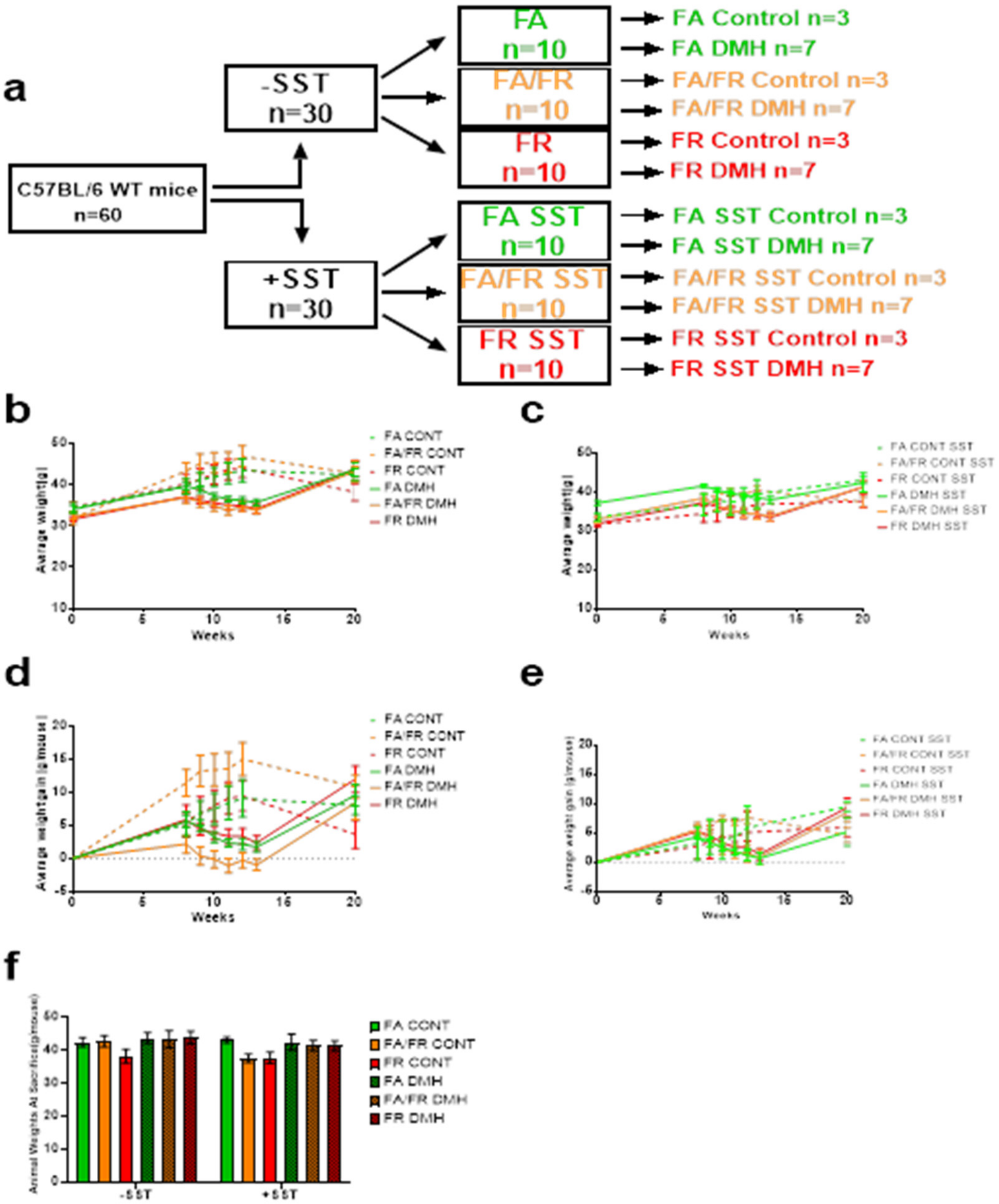

Figure S2. Effect of folate restriction on animal weights. a) Study design. b) Average mice weights fed diets without the sulfonamide antibiotic succinylsulfathiazole (SST). c) Average mice weights fed diets with the antibiotic SST. d) Average weight gain of mice fed diets without SST. e) Average weight gain of mice fed diets with SST. f) Mice weights at the time of sacrifice. Data is from mice fed: FA=folate adequate diet, FA/FR= folate adequate diet followed by initiation of folate restricted diet one week prior to DMH injections, FR= folate restricted diet. (SST= 1% succinylsulfathiazole added to diet), (CONT= Control; no carcinogenic treatment, n=3), (DMH= dimethylhydrazine; carcinogenic treatment, n=6-7) Bars, S.E.M.

Supplemental Figure S3

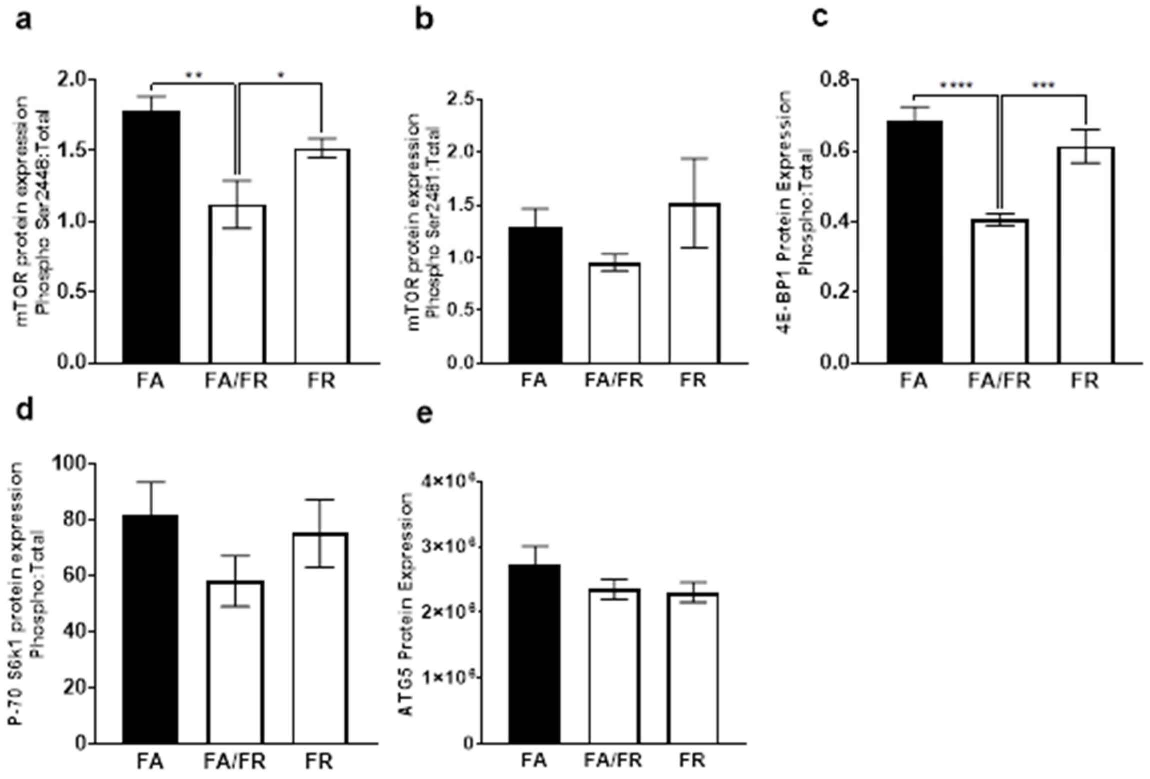

Figure S3. Folate restriction alters the expression of proteins in the mTOR signaling pathway in the liver of untreated control C57BL/6 mice. Western blot images and quantification of, a-b) mTOR phosphorylation at Ser2448 and Ser2481 residues, c-d) Phosphorylation of downstream targets of mTOR (4EBP1 (Thr37/46) and S6K1 (Thr421/Ser424)), and e) Expression of the autophagy related protein ATG5. FA=folate adequate diet, FA/FR= folate adequate diet followed by folate restricted diet for 14 weeks, FR= folate restricted diet for 21 weeks. Phosphorylated protein normalized to the respective total protein and expressed as ratio. Western images provided in supplemental table 1. Bands normalized to total lane proteins. Control animals were not treated with the tumor initiator dimethylhydrazine (n=3), Bars, S.E.M, *$p<0.05$, **$p<0.005$, ***$p<0.001$, ****$p<0.0001$.

.

Supplemental Figure S4

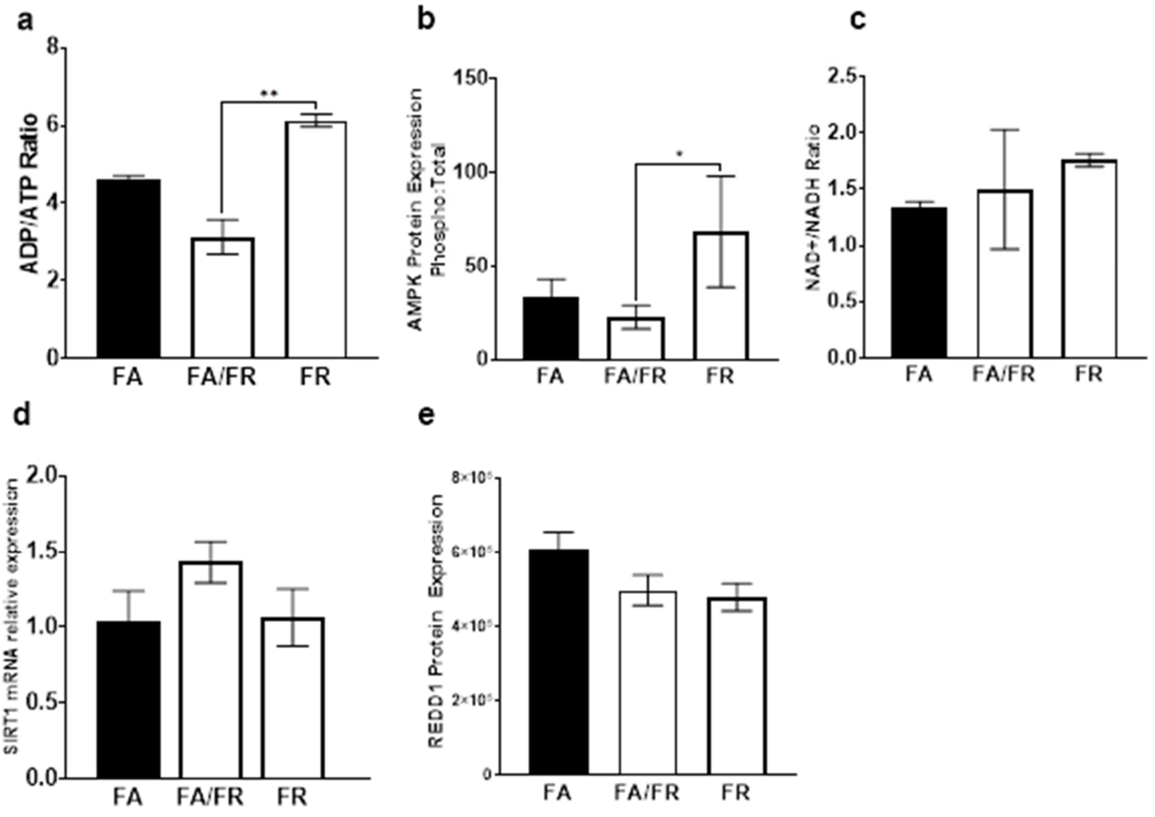

Figure S4. Effect of the timing and duration of folate restriction on energy levels and regulators of mTOR in the liver of untreated control C57BL/6 mice. a) ADP/ATP ratio in the liver of untreated control mice. b) Western blot quantification of AMPK (Thr172) protein phosphorylation. c) NAD+/NADH ratio. d) *Sirt1* mRNA relative expression. e) Western blot quantification of REDD1 protein expression. FA=folate adequate diet, FA/FR= folate adequate diet followed by folate restricted diet for 14 weeks, FR= folate restricted diet for 21 weeks. Westerns normalized to total lane proteins. Control animals were not treated with the tumor initiator dimethylhydrazine (n=3), Bars, S.E.M, *p<0.05, ***p<0.001.

Supplemental Figure S5:

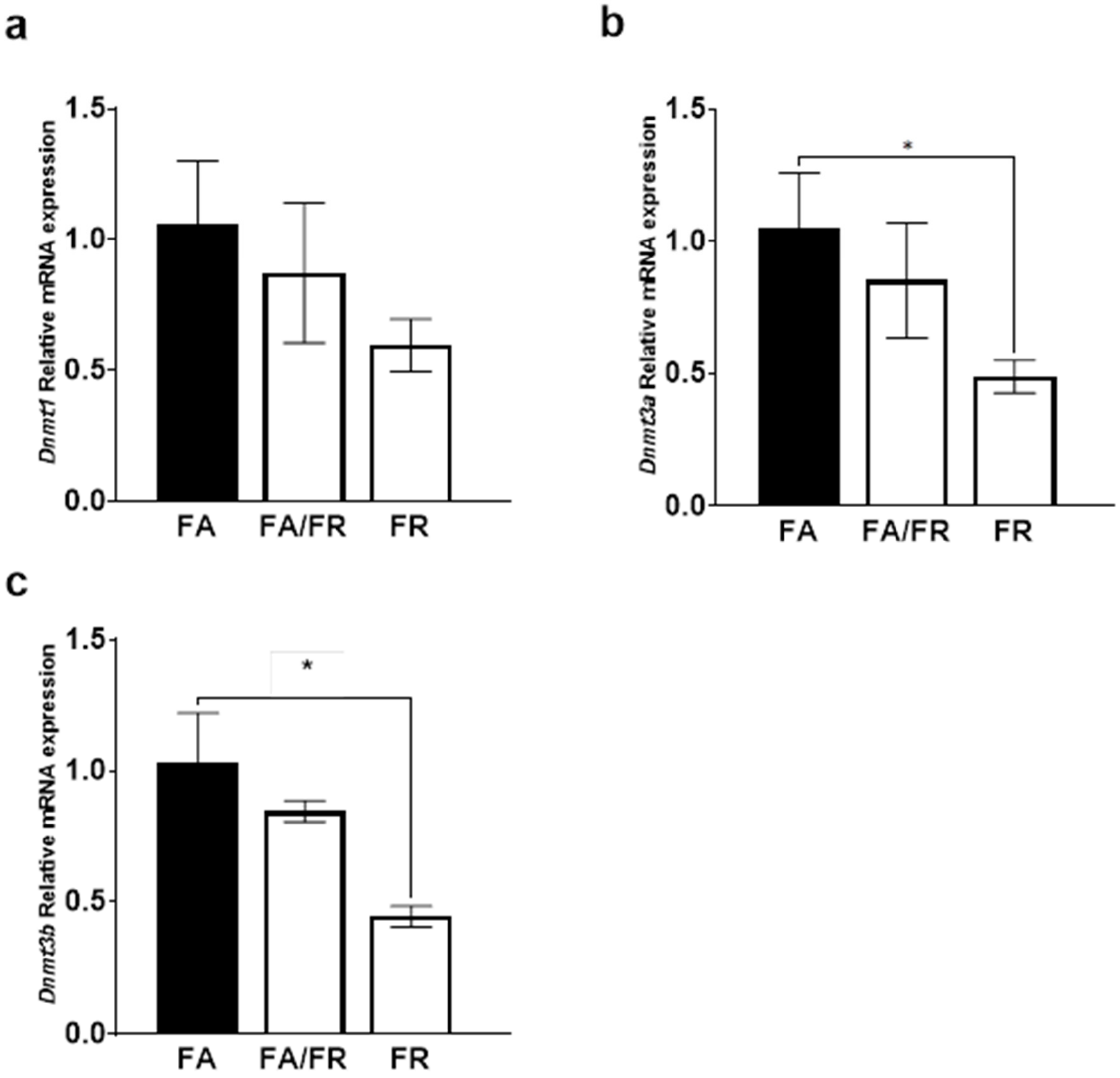

Figure S5. Folate restriction downregulates DNA methyltransferase gene expression in the liver of untreated control C57BL/6 mice. a) *Dnmt1* relative mRNA expression. b) *Dnmt3a* relative mRNA expression. c) *Dnmt3b* relative mRNA expression. Real time expression data normalized to geometric mean of *Gapdh* and *Rplp0*. FA=folate adequate diet, FA/FR= folate adequate diet followed by folate restricted diet for 14 weeks, FR= folate restricted diet for 21 weeks. Westerns normalized to total lane proteins. Control animals were not treated with the tumor initiator dimethylhydrazine (n=3), Bars, S.E.M, *$p<0.05$.

.

Supplemental Figure S6:

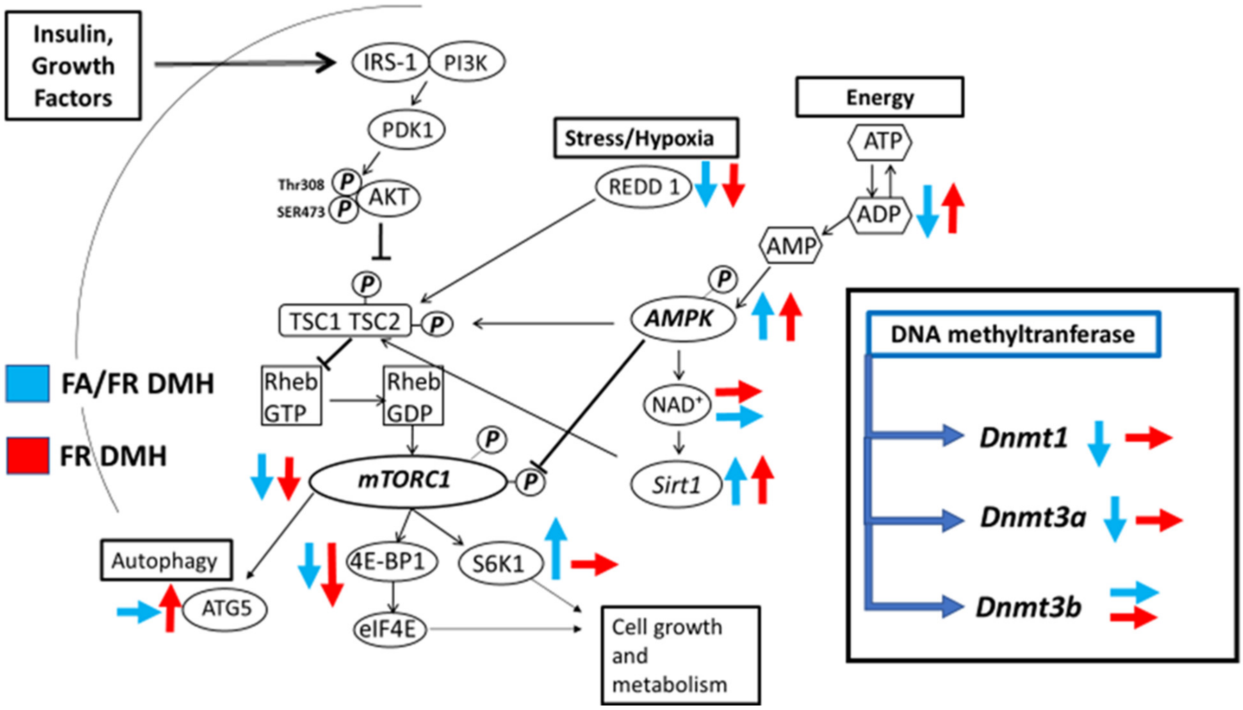

Figure S6. Summary of the effects of FA/FR and FR on the mTOR pathway and DNA methyltransferases in the liver of DMH treated mice. Arrow up or down denote significance compared to FA, horizontal arrows imply no significant difference.

.

Supplementary Table S1:

Table S1. Western Blots.

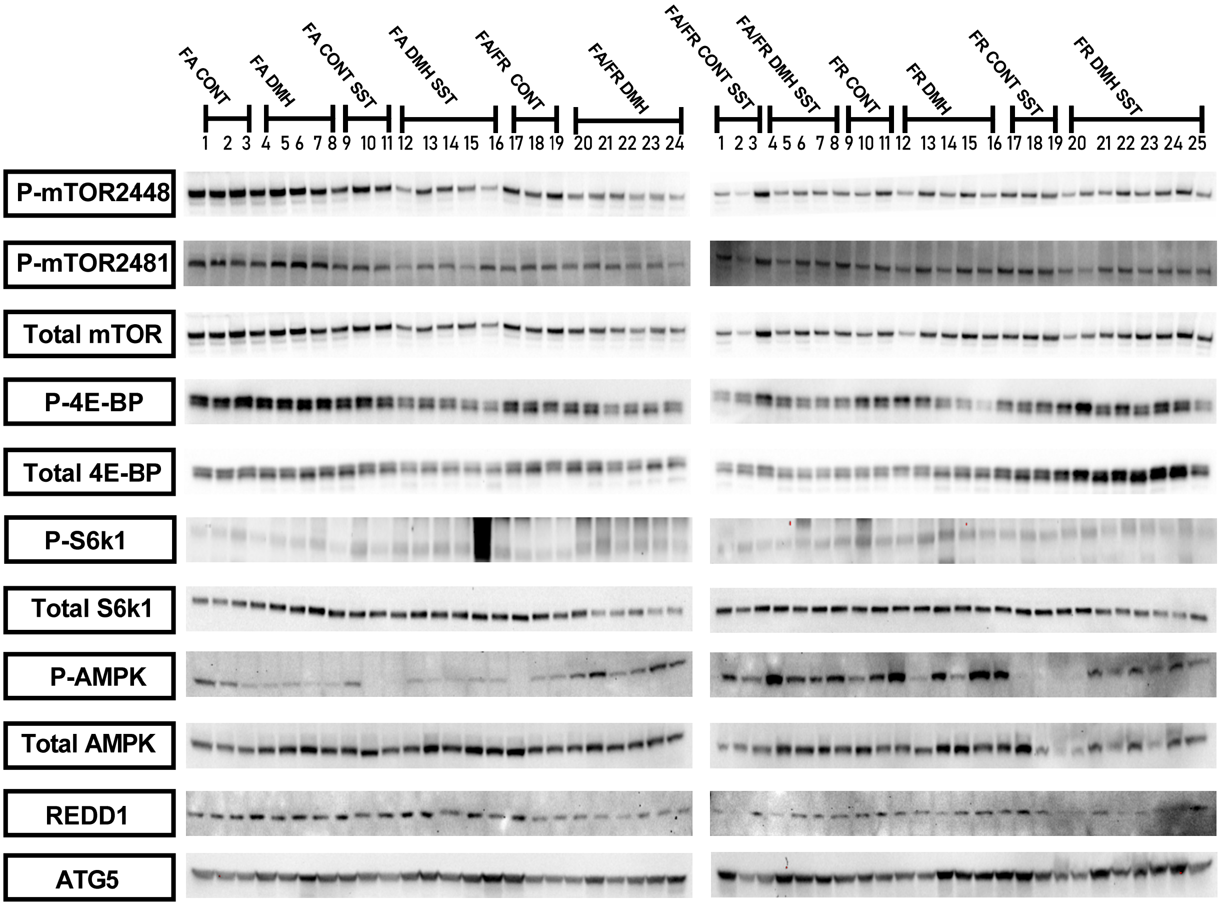

.

Supplementary Table S2:

Table S2. primer sequences.

Gene	Forward Primer 5′-3′	Reverse Primer 5′-3′
Gapdh	GCACAGTCAAGGCCGAGA	ATGGGGGCATCGGCAGA
Rplp0	GCTTCGTGTTCACCAAGGAGGA	GTCCTAGACCAGTGTTCTGAGC
Ung	AACAGGCTTGGTTGGCGAAGG	CGGCTGGCATCCTAAGACATC
P16	TGTTGAGGCTAGAGAGGATCTTG	CGAATCTGCACCGTAGTTGAGC
Sirt1	TGATTGGCACCGATCCTCG	CCACAGCGTCATATCATCCAG
Prdx6	AGCAGGTCCGTAGAAAGATCG	TCAAAGAGAGCCAGTCAGTAGG
Trx	CTGATCGAGAGCAAGGAAGC	TCATCCACATCCACTTCAAGG
Ogg1	CGGCTGGCATCCTAAGACATC	AACAGGCTTGGTTGGCGAAGG
Sod1	AACCAGTTGTGTTGTCAGGAC	CCACCATGTTTCTTAGAGTGAGG
Cth	GCTAGAGGCAGCGATTACACC	GCAGACATGAAGGTGTTATCTACAACC
Dnmt1	GGAAGGCTACCTGGCTAAAGTCAAG	GGGTGTCACTGTCCGACTTGC
Dnmt3a	TGGAGAATGGCTGCTGTGTGAC	CACTCATCCCGTTTCCGTTTG
Dnmt3b	AGTGACCAGTCCTCAGACACGAAG	ATCAGAGCCATTCCCATCATCTAC

Supplementary Table S3:

Table S3. Antibodies for western blot.

Antibody	Catalog number	Source
Phospho-p70 S6 Kinase (Thr389)	9205	Cell Signaling, Danvers, MA
p70 S6 Kinase	9202	Cell Signaling, Danvers, MA
Phospho-PDK1 (Ser241)	3061	Cell Signaling, Danvers, MA
Phospho-mTOR (Ser2448)	5536	Cell Signaling, Danvers, MA
Phospho-mTOR (Ser2481)	2974	Cell Signaling, Danvers, MA
mTOR (7C10)	2983	Cell Signaling, Danvers, MA
Phospho-4E-BP1 (Thr37/46)	2855	Cell Signaling, Danvers, MA
4E-BP1 (53H11)	9644	Cell Signaling, Danvers, MA
Atg5 (D5F5U)	12994	Cell Signaling, Danvers, MA
Phospho-AMPKα (Thr172)	2535	Cell Signaling, Danvers, MA
AMPKα	2532	Cell Signaling, Danvers, MA
REDD1 (DDIT4)	ab106356	Abcam ,Cambridge, UK

Supplementary Table S4:

Table S4. Average measured folate levels in serum, liver, and colon.

	**Control mice**			DMH mice	
**Folate level**	**Serum (fmol/ul)**	**Liver (nmol/g)**	Colon (nmol/g)	Serum (fmol/ul)	Liver (nmol/g)
FA	69.1	48.2	4.2	82.0	56.2
FA/FR	12.0	22.9	1.4	10.5	29.0
FR	4.0	21.9	1.3	5.5	24.5
FA SST	72.1	53.7	2.8	88.3	44.4
FA/FR SST	0.4	16.7	0.5	0.4	10.2
FR SST	0.4	8.7	0.3	-3.1	7.2
